# Abstracts, &c.

**Published:** 1889-01

**Authors:** 


### ABSTRACTS AND GLEANINGS.

### SOME OBSERVATIONS, AFTER 1,000 OPERATIONS FOR HÆMORRHOIDS.

Read in the Section on Surgery, at the Thirty ninth Annual Meeting of the American Medical Association, May, 1888.

JOSEPH M. MATTHEWS, M. D.
PROFESSOR OF PRINCIPLES AND PRACTICE OF SURGERY AND DISEASES OF THE RECTUM, KENTUCKY SCHOOL OF MEDICINE, LOUISVILLE.

Having for a number of years given special attention to the rectum, I have thought that this paper could be made more or less interesting by recording my individual experience in the operations looking to the relief of hemorrhoids. I have based my conclusions from operations done upon patients taken indiscriminately from hospital, dispensary, and private practice. Believing that an individual experience is worth much in forming an estimate, I give mine for what it is worth.

*Internal Hæmorrhoids.*—This I believe to be the most common of all rectal affections, though Mr. Allingham believes fistula in ano to be. In making this estimate I rule out the diagnoses made by patients, who usually pronounce any and all affections of the rectum or anus to be piles.

*Cause.*—With deference to our French *confrère*, I am satisfied that constipation is the chiefest among the causes of hæmorrhoids. Nor can I subscribe to the belief that through the dissections made by Verneuil we find sufficient evidence in the peculiar distribution of the veins, and the course they take in the coats of the rectum, to disprove the theory that constipation, sedentary occupations, drastic purgatives, prolonged use of enemata, etc., can institute true hemorrhoids. His idea that the superior hæmorrhoidal veins pass through "veritables boutonnières musculaires," and that these muscular button-holes have the power of contracting and causing such stasis and congestion in the superior hæmorrhoidal veins as to cause the "primmum mobile" in the formation of internal piles, I do not believe. Upon this theory was dilatation of the two sphincter muscles suggested as a cure for internal hæmorrhoids. Whatever might have been its success in France, I am sure it has failed of its object in America. This failure I attribute to the unscientific basis upon which it was practised. In a word, then, I believe hæmorrhoids to be veritable tumors, in the formation of which the arteries as well as the veins play a part. The mucous membrane of the rectum being movable, is everted at stool. Anything that impedes the return of blood will congest the part. This, sufficiently kept up, ends in the plastic deposit of inflammation constituting the material for the formation of piles. I quite agree with Verneuil, that the superior hæmorrhoidal veins are connected with the portal system, and in the main form internal hæmorrhoids, and that external piles are formed from the external and middle hæmorrhoidal which are not connected with the portal venous system, and hence the two venous systems, portal and general, are practically distinct at this point. This proposition is admitted, and yet we cannot admit the absolute separation of the portal and general venous systems. I have been thus explicit on this point from the fact that confusion has arisen over it.

*Sex.*—In my experience men are the oftenest affected with piles. This I account for on the basis that the portal circulation is in the male oftenest interfered with. This will outweigh the fact that child-bearing and displacements of the uterus are pregnant causes of piles in females.

*Age.*—A number of authors cite cases of piles in infants. I have never witnessed the affection in children under 10 years of age. Old age is mentioned as most favorable to the disease. This has not been my observation. Old people frequently mention that during adult life they were so affected, but as the years grew apace the piles disappeared. It is eminently a disease of middle life.

*Classification.*—The usual classification of piles is mistifying, to say the least. No anatomical difference can be drawn between the so-called venous and the arterial pile, and the commonly designated "capillary" pile frequently uncovers an artery of good calibre. The terms external and internal are quite sufficient for all practical purposes as applied to piles, for, as Mr. Erichsen says, "all internal piles should be cut off."

*Dangers.*—The only real danger from internal piles is hæmorrhage, and this can scarcely apply to any save the small incipient pile. The friction to which larger tumors are subjected causes such thickening of the mucous coat that bleeding, as a rule is impossible. Sometimes large hæmorrhoids prolapse and slough, hence require surgical interference, but this could only occur from an indiscretion.

*Operations. Injections of Hæmorrhoids.*—For a number of years the injection of hæmorrhoids with carbolic acid seemed to be a favorite plan with many in the proession. Being a discovery of the itinerants, it soon found favor with the laity, more because of its secrecy than from any merit. After the remedy was exposed, the profession was able to give it a fair trial. Many, for a while, were inclined to look upon it with much favor, for the reason especially that it was easily practised. After a few reported accidents, thoughtful men began the real study of the subject, the *modus operandi* of cure. After a sufficient length of time had elapsed for investigation, I took occasion to record my objections in an article read before the Kentucky Medical Society in 1878. In that paper the position was taken that the injecting of piles with carbolic acid was a dangerous procedure; that by the plan much inflammation was excited; the pain in the majority of cases very great and prolonged; that dangerous hæmorrhage was to be feared; that sloughing was the rule, hence ulceration, stricture, etc., might follow; that death might occur from hemorrhage, embolism, or septic trouble; that in no case could a radical cure be pronounced. After an elapse of ten years we find these predictions verified. Dr. Andrews, in his late work on "Diseases of the Rectum," p. 24, says: "The following accidents have been reported to us out of about 3,304 cases: Deaths, 13; embolism of liver, 8; sudden and dangerous prostration, 1; abscess of liver, 1; dangererous hæmorrhage, 10; permanent impotence, 1; stricture, 2; violent pain, 83; carbolic acid poisoning, 1; severe inflammation, sloughing and other accidents, 35." Of course this was an indiscriminate collection and the half was not told. The quacks would not confess their bad results, hence most of this table was from accidents and results so plain that the profession ascertained them. Allingham reports thousands of cases by ligature without an untoward symptom and no death. Others are of the same good report. Kesley, of New York, who a few years ago was inclined to advocate the injection plan, discountenances it now. Bell of Dublin, who has just issued an admirable work on diseases of the rectum, only says in a mention of it that the plan has been tried in America, but with poor success. The writer begs to say that what was said by him in 1878 he reaffirms in 1888. The remedy came in unawares, has been weighed in scientific scales and been found wanting. No authority to-day advocates its use.

*Dilatation.*—As has been suggested, after the views of Verneuil were promulgated, it was advised that dilatation of the sphincters be practiced for the cure of all internal piles. Such men as Gosselin, Dubrueil, Durch and others endorsed these views. The plan has been tried extensively in France and, the reports would indicate, with a moderate degree of success. In America it has proven a failure. The theory was wrong, hence the success bad. In the early formation of piles, I have no doubt but that good could be accomplished by the plan, but after a pile is well founded I am sure it could not be cured by any such procedure.

*Excision.*—It is a little surprising to hear Allingham say that he regards excision as one of our best operations for internal piles. I think that an author should consider the audience he is addressing, and surely this sentence could result in much harm. To the surgeon accustomed to handling the rectum, the danger might not appear so great, yet to the many that might try this operation it would be fraught with much danger. It certainly can never become a popular method. I have had the internes try it at my hospital clinics, and in each case great difficulty was experienced in controlling hæmorrhage.

*The Clamp and Cautery.*—This is generally known as Mr. Henry Smith's operation. It finds great favor in the hands of Dr. Kesley of New York. It occurs to me that after a surgeon has had a certain amount of success with any given operation in surgery, he is loth to give it up. This certainly must apply to Mr. Smith, who is a great advocate of the clamp and cautery for the cure of internal piles. That this operation, compared to others, is cumbersome, cannot be gainsaid. That it is attended with much danger must be admitted. Any clamp is but a temporary safeguard, and if a break is made in the seared edges of the wound, bleeding must of necessity take place, and we all recognize the great danger of hæmorrhage into the rectum after an operation has been completed. The method has found but little favor in this country, and I am satisfied that, by comparison with the ligature, it will fall into disuse.

*The Ligature.*—Certainly, of all known methods, the ligature stands preëminent as an operation for internal hæmorrhoids. It has stood the test of years in the hands of the most eminent surgeons. To-day it is the most popular method. Easy of execution, free of danger, and rapid in its results, it can but command the attention of all who are interested in this operation. To the principle involved in the use of the ligature all are agreed, but the method of application is to a degree disputed. The method, as practised by Mr. Allingham, is to dissect the hæmorrhoidal tumor away from its attachments, and then to surround the remainder, at its base, with a tight silk ligature. For the first few years in special practice I did the operation after this plan. I then modified it for the following reasons: 1. It was misleading to teach students after this fashion, because there was much danger in their dividing the artery which supplied the tumor. 2. There was more cutting than is necessary.

The modification consisted in running a delicate knife around the base of the pile, simply going through the integument. This saves any deep cut and at the same time removes all superfluous skin, or external piles. Indeed, there are many cases of internal hæmorrhoids which require no cutting at all. This point is not sufficiently brought out in the books. I allude to the large internal piles which have no complication as mentioned here, viz.: superfluous flesh, or external piles. These require only to be brought in sight, ligated and returned into the bowel. Much stress is laid upon the degree of tightness that should be accorded the ligature; some saying apply it loosely, others advising it to be drawn tightly. I am sure that the tighter a ligature is drawn the quicker and more affectual will be the cure. A point is made in suggesting the kind of material to be used in the ligature. Many prefer silk. I am in the habit of using the stoutest linen thread, such as is used by saddlers or shoemakers. The twist of the silk or other material, I am sure has nothing to do with it, as some seem to think it has. The kind of knot to be tied is spoken of, Mr. Allingham remarking that he ties the knot *three* times. Twice is quite sufficient, and the surgical knot has no advantage over the common hard knot. All internal piles existing should be ligated at the same sitting, and all returned into the rectrum. The greatest care should be taken in cutting off the tumors after ligating. It is much best to leave the whole mass, rather than to have one ligature slip after returning the tumors into the bowel. The presence of the mass in the gut cannot result in any harm ; the ligature is between it and the circulation. As a rule, then, it should not be cut off. A number who have written upon this subject say that the bowels should be confined for from five to seven days, and a light liquid diet enjoined. Certainly this is a mistake, for two reasons: 1. In this length of time the fæces will become hardened and impacted. 2. These patients generally require all the nourishment they can get. When following this advice I have had as much or more trouble result from impaction than from the original operation. My habit is to purge the patient the day of the operation, and to give an aperient on the second day after the operation, and each succeeding day thereafter until the patient is discharged. Consequently a full diet is prescribed if required. When operating under an anæasthetic I always divulse the sphincters, more especially if any cutting is done. This prevents the contracting of the muscles, and obviates much pain when inflamed.

*Anæsthetics.*—It is often asked if the operation for internal hæmorrhoids can be done without the use of anæsthetics. If the sphincter must be divulsed because of the complication of fissure, ulceration, or what not; or if any cutting is to be done, then an anæsthetic is absolutely necessary. If internal hæmorrhoids exist without any such complication, and protrude well in response to the bearing-down effort, then anæsthetic is *not* necessary. It is often suggested that cocaine could be used with benefit in these operations. In the removal of external piles much benefit is derived from throwing the solution under the growth. In the operation for internal piles I have found it of little value.

*Antiseptics.*—Unfortunately, strict antisepsis cannot be practised in these operations. Fortunately, it is not as necessary as when operating elsewhere. I am in the habit, however, in all these operations, of having strict surgical cleanliness as regards both the person and instrument. If any cutting is done the parts are dusted with iodoform, and the gauze of the same applied over the wound.

*Results.*—As the caption of this paper intimates, I have operated about one thousand times for hæmorrhoids by the ligature. I have never had to operate the second time upon the same patient for the affection. Have never had an unnatural contraction around the anus as the result of the operation, nor had ulceration or stricture to result. I have had in this time one case of tetanus, which I believe to have been superinduced by a debauch, the patient having been drunk for several days before the operation. The tumors protruded, strangulated and mortified, hence the operation. He recovered from the tetanus under the bromide treatment. Have had one case of secondary hæmorrhage occurring on the third day. The rectum was plugged and the bleeding stopped. Also one dangerous case of hæmorrhage which occurred one hour after operation was done, in consequence of the slipping of the ligature, the pile having been cut off.

The patient was pulsless and cold when seen, but the artery was quickly secured and tied, and he made a good recovery. I have never had a single death result from the operation, and but few untoward symptoms.—*Jr. Am. Med. Ass.*

---

### LARYNGOLOGY AND RHINOLOGY.

UNDER THE CHARGE OF

CHARLES E. SAJOUS, M. D.,

Lecturer on Laryngology and Rhinology in Jefferson Medical College, Philadelphia.

---

### HYPNOTISM IN DISEASES OF THE NOSE AND THROAT.

Schnitzler, of Vienna, in a case of paralytic aphonia (a girl of twenty) in which he had employed galvanism and all other measures usually resorted to without success, induced hypnotism by directing the light of his head reflector into the patient's eyes. Dyspnœa, which had also been a prominent symptom of the case, immediately disappeared, while the voice was found to be normal and strong when she was awakened. The improvement lasted a few days, when the symptoms returned, to finally yield altogether, however, after a few *séances* of hypnotic sleep. In several other cases of the same kind, and in a stubborn case of spasm of the glottis, Schnitzler states that he was equally successful.

A very interesting case was that of a lady of twenty, in which laryngeal chorea accompanied general hemicrania. Hypnotism, induced as in other cases, caused immediate cessation of the twitches of the face and larnyx and the jerks of the limbs. The benefit being of brief duration, "suggestion" was added to the hypnotic sleep, resulting in a permanent cure.

In another case, one of the nasal polypi, hypnotism was accidentally induced by one of his pupils by means of the frontal mirror. Several of the growths were snared away and their seats of implantation cauterized. Upon being awakened the patient was not aware of the operation, having suffered no pain whatever. Some time later, there being occasion to again operate, she was intentionally hypnotized, again undergoing the operation without the least suffering. This case, according to Schnitzler, was in nowise hysterical.—*International Klin, Rundschau*, August, 1888.—*Satellite.*

THE CREOSOTE TREATMENT OF PULMONARY TUBERCULOSIS.—Sommerbrodt, of Breslau, is quoted by the *Medicinische Chirurgische Rundschau* for June, 1887, as reporting five thousand cases of pulmonary tuberculosis treated by creosote. His studies have extended over nine years in time, and the patients so treated were all ambulatory cases. By the use of Bouchard's and Gimbert's formulæ he treated a number of patients, twenty-seven per cent of whom recovered. The formulæ most used were 13.5 parts creosote to 1 quart alcohol or Malaga wine ; also 2 parts of creosote to 150 of ol. morrhuæ. Of both of these preparations from 3 to 6 minims of creosote were taken daily. He also used the following : Creosote, 3.5 parts ; alcohol, aquæ, ää 125 parts. Of which a teaspoonful was taken twice daily in a glass of water by thirty patients. Following the method of Reuss, the best results were given by the following : Gelatin capsules, containing each 3-4 minim of creosote and 3 minims of balsam of tolu. The first day of treatment the patient took one of these capsules, on the second day two, and then for eight days three were taken after meals, with water. In the second week four capsules daily were taken ; in the third five capsules daily ; and in the fourth week six capsules daily, always after the principal meals of the day. The remedy when so given, was generally well borne ; they were rarely taken except at meals ; their use was continued frequently for a year, and was combined with treatment in favorable climates. The best results were obtained in young patients in the first stage of the disease, with ill-defined symptoms. In scrofulous gland-affections the results obtained were also good. The irritation and frequent cough present were generally relieved, so that narcotics could be discontinued ; bronchial secretion and night sweats lessened. Summerbrodt believes that many such cases can be greatly improved. The remedy must be used for three months or a year, and the more that can be taken the better the results.—*Cincinnati Med. Jour.*

---

**Ovaries not Essential to Sexual Appetite.**—Lawson Tait said at the British Gynecological Society, that he had seen a number of cases of women whose ovaries had been removed while they were yet virgins, and in whom no lack of the sexual appetite was experienced. Still, quite as remarkable was the evidence he had obtained from three virgins in whom the uterus, ovaries and tubes were removed without an influence upon the sexual desire.

### DIPHTHERIA AND SCARLET FEVER.

I have found Listerine very valuable in the management of diphtheria as a gargle, but most frequently as a spray (diluted with about four or five parts of water); it cleanses the diseased surfaces and removes the unpleasant odor and taste of the secretions from the throat of the patient. In nasal diphtheria (and pharyngeal as well) by adding to hot water and applying with a syringe through the nostril, (using gentle force only), directing the patient, if old enough, to understand, to hold the accumulating fluid in the throat for a moment, and then expel it. If it should be accidentally swallowed even in liberal quantities, no harm will result, but benefit will accrue to the sensitive alimentary tract. If the patient should unfortunately be incapable of acting upon the instructions given above, a few drops may be applied to the nostrils by means of a medicine dropper and thus allowed to slowly percolate over the inflamed surface of the nasal and pharyngeal mucous membrane.

To secure fluidity and free exit of discharges from the nose, melted vaseline (incorporated with boracic acid) may be applied in the manner above indicated in conjunction with the Listerine. Any one who has witnessed the demoralizing effects of the accumulated secretions in nasal and pharyangeal diphtheria, can appreciate the value of remedies which obviate or mitigate the trouble.

I consider Listerine of very great value in scarlet fever, as well as in diphtheria, and for similar reasons.

As an aid in the securement of the personal prophylaxis of scarlet fever cases, it is my practice to have the patient sponged off daily with diluted Listerine. By such procedure the question of contagion is almost eliminated during the desquamation period, and, in consequence of the component parts of Listerine, it relieves the itching and irritation of the skin, so frequently present, induces a refreshing after-effect, thus adding to the personal comfort of the patient, a matter of no small importance.

I. N. LOVE,
St. Louis Hospital.

---

**The Antagonism of Cocaine.**—Cocaine continues to attract much attention in Medical circles, and many new facts concerning it are being discovered and reported. According to recent German investigations, the drug has "a stimulating effect on the psychic and motor nerve-centres, increases the rapidity of breathing, quickens the heart's action, and promotes chemical change going on in the tissues." From its action on the nerve-centres, it is declared "the best of known stimulants." In this respect it is in marked antagonism to chloral, which "depresses the functional activity of these centres." If an animal has been narcotized by a dose of chloral that would otherwise prove fatal, it can be roused and saved by the injection of a small dose of cocaine.

In regard to temperature, however, this antagonistic action fails. It is well known that chloral tends to lower the animal temperature, while on the other hand, cocaine tends to raise it. In some instances a rise of 4° C. in the course of half an hour has been observed; but, after poisoning by chloral, this effect of cocaine is not produced. In all other respects the antagonism of the two agents is complete, dangerously large doses of chloral proving harmless after the administration of small doses of cocaine. It is suggested, however, that as the first effect of cocaine, notwithstanding its subsequent action as a stimulant, is to depress the respiratiory function, it is advisable, in cases of chloral-poisoning, to induce artificial respiration for a short time after administering the cocaine.

Cocaine is also antagonistic to chloroform and ether, and inhalation of either of these agents will allay the convulsions due to poisonous doses of cocaine. The severer symptoms having been thus allayed, chloral may be afterwards given to keep up the effect. On the other hand, cocaine may be used as an antidote in cases of poisoning by narcotic agents, especially such as cause great depression of the respiratory and cardiac centres.

It may be added, that cocaine is found to be a perfect substitute for strychnine, as it has all the therepentic activity of that drug, without any of its poisonous qualities.—*Pop. S. News.*

---

**How to Treat the Eye with a Cinder in it.**—R. W. ST. CLAIR writes the *Medical Summary* as follows:—

"Nine persons out of every ten with a cinder or any foreign substance in the eye, will instantly begin to rub the eye with one hand, while hunting for their handkerchief with the other. They may and sometimes do, remove the offending cinder; but more frequently they rub until the eye becomes inflamed, bind a handkerchief around the head, and go to bed. This is all wrong. The better way is, not to rub the eye with the cinder in at all, but rub the other eye as vigorously as you like.

"A few years since, I was riding on an engine. The engineer threw open a front window, and I caught a cinder that gave me the most excruciating pain. I began to rub the eye with both hands. 'Let your eye alone, and rub the other eye [this from the engineer]. I know you doctors think you know it all; but if you will let that eye alone, and rub the other one, the cinder will be out in two minutes,' persisted the engineer. I began to rub the other eye; and soon I felt the cinder down near the inner canthus, and made ready to take it out. Let it alone and keep at the well eye," shouted the doctor *pro tem.* I did so for a minute longer, and looking in a small glass he gave me, I found thc offender on my cheek. Since then I have tried it many times, and have advised many others, and I have never known it to fail in one instance (unless it was as sharp as a piece of steel, or something that cuts into the ball, and required an operation to remove it). Why it is so, I do not know; bnt that it is so, I do know, and that one may be saved much suffering, if they will let the injured eye alone, and rub the well eye."—*Pop. Sci. News.*

**Hot Mustard Baths in Pneumonia.**—Dr. Weber, of New York, in American Journal of Obstetrics says: I attended Jennie A—— aged 10 months, previously a healthy and robust child, afflicted with extensive pneumonia, after having been sick for a week with bronchitis. On the third day after I had seen and treated her in the usual manner, she became rapidly cyanosed and died.

One year after I had treated Jennie A——, another female child of about the same age, and similarly good constitution, in the same family, became affected in the same way, and I recognized at once that there was pneumonic infiltration in both upper lobes. In spite of emetics, digitalis, mustard-plasters and poultices over chest, she became cyanotic on the third day, with stertorous breathing, cold extremities and failing heart's action. It occurred to me at this stage to immerse the patient in a hot mustard-bath of 105 degrees F., prepared by mixing a pound of ground mustard in a baby-tub full of hot water. I kept her in it ten minutes, making thorough friction all over the surface, until the skin was pinkish. After being put to bed, which I had previously well warmed, the child began to breath easier, and soon fell asleep. The skin remained warm and an hour after the bath she was perspiring freely. The pulse now became stronger and less frequent, and the child took the breast readily.

I repeated the bath in four hours with the same good results. In all, I administered five mustard-baths in forty-eight hours, when I had the satisfaction of seeing my patient convalescent, and that, too, without any medicine whatever.

Case II. J. O——,female child six weeks old, had had capillary bronchitis for some days, and was almost moribund and pulseless when I saw it. Mustard-baths were administered every three hours and the child rapidly recovered.

Case III. G. G——, boy aged four months; broncho-pneumonia; pulse 160; cyanotic and sinking fast. Gave hot mustard-baths. Child was out of danger after four baths, within thirty-six hours.

Case IV. I. S——, girl six months old; broncho-pneumonia; cyanotic; comatose and almost pulseless. Recovered by the use of the baths.

Case V. Geo. C——, aged 8 months; broncho-pneumonia, following measles; condition hopeless before baths were used. Gave seven baths in two days. Recovered rapidly and perfectly.

Case VI. Girl, seven years old; had been sick about two weeks when I saw her, with Dr. R. At that time her temperature was 103° respiration, 72 per minute; pulse, 140. Our prognosis was unfavorable. Gave hot mustard-baths and she recovered completely.

I have employed the hot mustard-bath successfully, when the patients were at their worst, and have succeeded in relieving the conjested lungs and helping the over-burdened heart, after all other remedies had failed.

DR. J. E. CLARK, of Brooklyn, has been successful in treating incontinence of urine in children by dilating, once in a while, the urethral canal with the ordinary sound.

**Artesian wells in Memphis.**—The cities of the Mississippi valley have never been noted for for the purity of their water supply, as they have depended largely on river water. Recently the city of Memphis has been experimenting with artesian wells, and has found an inexhaustible supply of the best water directly under its site. A true artesian basin, covered by a perfectly impervious stratum has been discovered, which is now fast displacing the unsatisfactory Wolf river as a source for water. Hitherto this river has supplied the city's wants. Thirty-two artesian wells have been driven over an area 2,000 by 300 feet. They are driven to a depth of about 450 feet. They first pass through 20 feet of bluff loam, then through 24 feet of sand and gravel, and finally through 150 feet of hard, impervious clay. The water bearing stratum is then reached, which consists of perfectly clean sand seven hundred feet deep. The water rises far above the level of the Mississippi River. Permanent works are now in progress. A large well is to to be sunk 80 feet below the surface. From this a horizontal tunnel 2,000 feet long will be carried through the hard clay. This tunnel will be five feet in diameter, and the wells will be connected with it. The water will be pumped from the large well. The tunnel can be extended indefinitely, and more wells can be bored as the supply may need extension. The temperature of the water is practically uniform, and averages about 62° Fah. It is impossible to overestimate the importance of this development as far as Memphis alone is concerned. But the same basin includes many other cities, and eventually a large area may be benefited by the discovery so happily made at Memphis. Mr. R. C. Graves, manager of a local ice company of that city, had used artesian water for making ice, and to him is largely due the credit for the new Memphis water supply.—*Scientific American.*

---

**Chloroform in Labor.**—A case reported in the October number of the *Chicago Medical Times*, by Dr. E. E. Curtis, in which in a twin labor, several days elapsed between the births of the children, during which time the os kept firmly closed, calls to mind a case in my practice which I will relate:

Was called in haste to assist in a case of labor, colored woman, the old granny sending word that she could not see "head nor tail" to it. The patient, a multipara, it was stated to me on arrival, had been in pretty hard labor from two o'clock, a. m. till five p. m., when I saw her, and that there had been no advance. Found slight bearing down pains.

Not wasting time to make further inquiries, "I pulled off my coat and rolled up my sleeves," expecting that this "Jordan" would be "a hard road to travel." I "waded in," found patient's pelvis of ample capacity, as well as the avenue leading therein. Soon detected the head presenting, quite loose, and movable in any direction. Passing my fingers further on I discovered that the os had firmly and closely contracted around the child's neck. This seemed to have been the only impediment to a delivery that should have been completed hours and hours before.

Knowing that ergot drives but not relaxes, I began the administration of chloroform, as the chosen agent, and no sooner than the patient began to be impressed by it, she had a pain or two which completed the labor.

I can't explain these occurrences, except by supposing that nature acted without co-ordination of movement, as she sometimes does in cases of retained placenta.

To obviate this last-named trouble the removal of the secondines sufficiently soon after the exit of the fœtus is, generally, all that is necessary.

In a number of instances I have noticed that, after so long a time succeeding fœtal delivery, the os uteri has a tendency to close up and retain the afterbirth, unless some means are used to cause its expulsion. The pains or contractions for the discharge of the placenta seem to need coating anyway.

Quite recently I reached a woman who had been delivered of her child six hours before I saw her, but the afterbirth was retained in spite of all the tomfoolery of the old granny; hence my visit. I learned that she had had no pains to expel it. Fortunately there was no hemorrhage. Upon passing my finger, I found the os firmly closed, not admitting a finger without effort, which caused much pain. I immediately gave her chloroform and soon extracted the placenta, and all did well.

I cannot speak too highly of the usefulness of chloroform during the pangs of labor. I do not use it in every case, but in all of those where there exist great sensitiveness and evident suffering. I use it in such cases.

It is very often that nervous women suffer very much from cramp and "false pains"—pure torture—which do not in the least contribute to the advancement of labor—nay, rather retard it. To such the administration of the *Magnum Didonum* is simply an act of humanity. My plan of using it I will give, but claim no originality:

Take an ordinary tumbler and place a linen rag in the bottom, six inches square and folded upon itself five or six times. Then take a piece of white oak split and cut it of such length that when pushed down to the bottom of the glass it will stay bent and prevent the rag from falling out when the tumbler is inverted. Pour on the rag thirty or more drops of chloroform, tell the patient to close her eyes, and apply the tumbler over nose and mouth, resting upon them. She alone must hold the glass and breathe *ad libitum*. Before harm can be done she will let the glass fall from her hand upon the bed. She must not let the glass rest upon the pillow, but hold it in her "hand." She should not be allowed to take chloroform to the extent of putting her sleep. The amount of chloroform is to be renewed from time to time, and more than the quantity stated may often be required. Thus used, so far from labor being retarded by the chloroform, it decidedly hastens it, removing all obstacles, mental or corporeal.

Magnolia, S. C. J. M. SANDERS, in *Medical Brief*.

**The Medicines that can Safely be given Nursing Mothers.**—A very useful series of experiments has been made by professor Fehling (*Medical Press*, May 9, 1888; *Therapeutic Gazette*), in order to determine the drugs that may safely be given to nursing mothers. His first conclusion is the very sweeping one, that "all soluble agents, when ingested, may pass into the circulation and be excreted in the milk." An apparent exception to this rule, however, exists in the case of the ferrocyanide of potassium. Among the drugs tested were:

1. Salicylate of sodium, which was found dangerous to the infant in doses (to the nurse) of 45 grains daily.
2. Iodide of potassium, a drug slow in its excretion, and one that may be safely given in doses of 3 grains daily. Such a dose may, however, we are sure, be greatly exceeded in many cases.
3. Iodoform is gotten into the system of the child more easily through the nurse than by giving directly to the child. Even when the wounds of the mother were dressed with iodoform, the drug was observed in the urine of the child.
4. An important statement, if true, is that mercurial salts given to the mother do not affect the infant at all, or at least very slightly.
5. Experiments with opium and morphine convinced Prof. Fehling that 25 drops of tincture opii (German Pharmacopœia), and $\frac{1}{10}$ to $\frac{3}{10}$ grain of morphine, could be given safely to the mother.
6. Chloral, he found, could be given in doses of 23 to 45 grains.
7. Atropine very easily affects the child, even in small doses.—*Medical Chips.*

---

[From Boston Medical and Surgical Journal.]

**Uses of Gelsemium Semper-virens.**—Dr. G. M. Garland says: The dose of gelsemium depends upon the preparation used and the effect which one desires to obtain. For the relief of neuralgia one should give 3 to 5 drops every half hour or hour, according to the intensity of the pain. To produce sweating, 1 drop every half hour is sufficient, provided the patient be well wrapped up in bed; 1 drop of the fluid extract will relieve the cough and discomfort of acute bronchitis; the tincture of gelsemium sempervirens is slightly weaker than the fluid extract.

The advantages which gelsemium sempervirens can legitimately claim,

1. It has an agreeable taste, and is not repulsive to adult or child.
2. It does not irritate the stomach or bowels.
3. It produces no depressing afte effects from ordinary doses, the sleep is natural, and the patient awakens refreshed.
4. In ordinary doses it causes no depression of the heart, and it can be used in all forms of organic diseases of the heart.
5. It does not create a habit. There is no depression of nerve centres following its use, and therefore no craving for more cf it.
6. Its toxic symptoms are very characteristic and striking, and they appear early, so that plenty of warning is given.

Morphine is the best antidote, combined with digitalis and artificial respiration.

**The Local Treatment of Diphtheria by Vleminck's Solution or Sulphuret of Calcium.**—GEORGE E. HUBBARD, M. D., of New York says: It occurred to me nearly two years ago that if the Sulphuret of Calcium is so effective in destroying the itch-mite, it would serve a good purpose in diphtheria, and I therefore tried it, at first carefully, with a camel's-hair brush, on my next case, to my great satisfaction. I now use the clear solution undiluted, by means of a spray, every half hour until the disease is under control, and then at longer intervals. In cases of very young children it may be best to add a little water to the solution at first until we are satisfied that it does not irritate the tender mucuous membrane, but I have almost invaribly used it pure, and have met with such marked success that I use no other local application.

In certain cases, every member of a family has been affected at about the same time, which would indicate that the diagnosis was corect.

It is not to be understood that this is all I do in the treatment of diphtheria, although in many cases I think this would suffice. I always make a practice of thoroughly fumigating the apartments with sulphur, and use such medicines as may judgement dictates. The principal thing to do is to destroy the disease-germs as early as possible.

The solution is prepared as follows:

Take, of lime, one part; sulphur, two parts; water, twenty parts. Shake the lime with some of the water, then add the remainder and the sulphur; boil to twelve parts and filter.

Under the use of this solution in spray, even sparingly applied, the diphtheritic patches undergo a change in a few hours. The temperature soon subsides, and a general improvment in the condition takes place almost from the first application. In some cases the patches disappear entirely in a day. If the false membrane has developed rapidly before the physician has seen the patient, under the influence of the spray it will be effectual even then in arresting systemic poisoning, and sooner or later the tough membrane will detach itself. Do not by any means allow the patient to swallow any portion of the false membrane. By gentle manipulation it can sometimes be removed without causing any irritation.

I have no doubt that the solution under consideration does much toward preventing constitutional infection as it is taken into the patient's stomach. Its stimulant, laxative, and somewhat diaphoretic and diuretic action are quite marked.—*N. Y. Med. Rec.*

**The Relation of Masturbation to Stricture of the Urethra.**—S. W. Gross, of Philadelphia, as a result of a clinical and statistical study of three hundred and thirty-one cases of masturbation, concludes, emphatically that urethral stricture may be expected in nearly nine-tenths, of all masturbators who have never had gonorrhœa, and that, as a rule, the stricture will be found to be of large calibre, single and seated near to the meatus.—*Med News*, Sept. 29, 1888.

**Thuya Occidentalis Epithelioma of the Larnyx and Pharynx.**—Baratoux, of Paris, reports encouraging results in cancerous growths, especially epitheliomata, from the use of thuya, administered internally and locally. He tried the tincture and alcoholic extract of both *thuya occidentalis* and *thuya orientalis*, but the latter in no way resembled the former in therapeutic value.

He administered daily doses of twenty drops of the tincture, which were gradually increased until forty-five to sixty minims were reached. At the same time the diseased parts were painted with the pure tincture every second or third day, sometimes every day. If the trouble be located in deep parts, such as the post-nasal space, he recommends a spray composed of the following ingredients :—

℞ Tinct. of thuya occidentalis, . . . gr. lxxv.
Distilled water, . . . . . . . ℥iij.
Glycerine, . . . . . . . . ʒiiss.

The effects of this medication are very rapid, eight days at most sufficing to cause diminution, and even disappearance, of the fetidity of the mucoid and purulent expectoration. The secretions also become less abundant, and about the twentieth day one may observe marked change in the volume of the vegetating mass.

A remarkable fact is that in papillomata of the nose and ear, as if the structure of the tumor be mixed, the neoplasm diminishes in volume, but cannot be made to disappear completely. Sarcomata and carcinomata do not seem to be influenced at all by the drug.—*Journal de Médecine de Paris*, May 13, 1888.

**Hypnotism at Berlin.**—A certain Dr. Feldmann, who is not, we believe, a qualified medical man at all, has been exhibiting his attainments as a hypnotizer and mind reader before the Berlin Medical Society. He showed all the familiar performances of "subjects." It is interesting to note the progress of medical opinion. Dr. Feldmann could not get before a reputable society when in this city last winter; but in Berlin he has appeared before the leading medical society, presided over by Professor Virchow.—*N. Y. Med. Rec.*

**Eruptions Due to Antipyrine.**—Spitz has collected references to fifty-two cases of antipyrine eruptions. In forty-one cases the eruption is described as rubeola-like, in four urticarian-form, in others erythema-form, or papular. The antipyrine was given in thirty cases of typhoid fever, in eight cases of phthisis, in two cases of articular rheumatism. In the other cases the concurrent disease was not reported. The seat of the eruption varied greatly in different cases, but was usually on the extensor surface of the limbs. The average duration of the eruption was four to eight days.—*Ann. de Derm. et de Syph.*, vol. ix, 1888, p. 192; from *Therapeutische Monatshefte.*

**The Menses.**—Give iron when the menses are scanty and lack color; give arsenic when the flow is too profuse, prolonged, or frequent.

[From Kansas City Index.]

**For Recent Prolapsus Uteri.**—In the treatment of recent cases of prolapsus, there is no method that gives more prompt and satisfactory results than this : An injection of very hot water is first to be given ; in this water may be dissolved borax and tannic acid in proper proportions. The hot water injections are of course most beneficial in the stage of inflammation which frequently follows prolapsus, but even in cases where there is no trace of inflammatory trouble they will prove of much advantage, exercising a soothing influence, and, if containing the drugs, an astringent effect. After the injection, a tampon of cotton, saturated with glycerine containing a small amount of tannic acid, should be introduced, compressing the engorged veins, and lifting the uterus to its normal position. The injection should be used night and morning, and a foesh tampon introduced by the patient after each injection. If this is kept up for a considerable time, under the supervision of the physician, the very best results may be expected.—*Medical Chips.*

---

[From the Medical Press.]

**Chloride of Ammonium in Neuralgia.**—W. T. Greene, M. D. : Three weeks since a lady called at my house, when her husband, who accompanied her, told me that he had been suffering from neuralgia in the head and neck, left side, for fifteen weeks without a day's intermission, and that the pain was getting worse instead of better, so that, at times, he felt as if he must "go out of his mind." He said that he had been prescribed for by several medical men, whose names he mentioned, with scarcely any alleviation, even temporary, of his sufferings. He produced a bundle of prescriptions which all rang the changes upon sulphate of magnesia, quinine and iron, in various more or less elaborate combinations. "They have not done me a bit of good," he said, emphatically; "can you give me anything else?" I made him up a mixture of chloride of ammonium, twenty grains to the dose, which he took away with him. The next evening the gentleman called to say that he had taken what I had given him, and, for the first time for fifteen weeks, had passed a day without pain, having felt an improvement after taking the first dose of medicine. He begged for another bottle, as he was afraid the neuralgia might return, so I gave it to him, but he did not take the whole of it, and has had no return of the neuralgia since. Chloride of ammonium is a very simple, most valuable and strangely neglected drug, which I have never known to fail in the treatment of neuralgia.—*Medical Abstract.*

**Treatment of Erysipelas.**—Nussbaum recommends the application of a pomade of equal parts of lanolin and ichthyol as a very speedy and simple method of cure in erysipelas. He covers the parts with salicylated cotton, and obtains a painless cure usually in two or three days.—*N. Y. Med. Rec.*

**The Treatment of Typhoid Fever by Carbolic Acid.**—Sidney Gramshaw (*Lancet*, 1888, page 1248) reports his results with this drug, used after Roshe's method, in one hundred and sixteen cases during the last seven years. The general management of the cases consisted in the administration, principally, of a diet of milk and of a mixture consisting of one and a half minims of pure carbolic acid and two minims of tincture of iodine every four hours for the first fortnight, or until the urgent symptoms abated, then three times a day. The good effect is manifested almost immediately, and it sometimes happens that a case is cut short almost as quickly as acute rheumatism by salicylic acid. Brandy and champagne are given if needed. Beef tea is avoided during the fever, as it is apt to produce diarrhœa. If the medicine produces vomiting the dose should be diminished. Only one of the one hundred and sixteen cases died, and this of pneumonia after the fever had disappeared.—*Amer. Jour. Med. Sciences, Sept.*, 1888.

---

**Croup.**—Dr. John Cook, of Utah claims great success in the treatment of croup and says: (in *Med. World*) I dispensed with all emetics and expectorants as worthless, and applied myself to the nature of the disease. I came to the conclusion that as a fever always exists in true croup, my attention would have to be engaged immediately to procure a remedy to conquer it. What could I do better than to fall back upon the noble remedy, aconitum napellus, as a weapon of defence? Very well; so far, so good. One step gained in advance; but another attack was made upon me by the inflammatory action of the mucous membrane, with the exudation of a false membrane upon it. Here I made a halt, and began to look around me for a remedy that governed that part. My choice fell upon spongia usta as the remedy, which worked like a charm, leaving me nothing to contend with but the croupy cough, for which I gave hepar sulph. This last attack brought me out of the fight crowned with victory.

FORMULA:

| | | |
|---|---|---|
| ℞ | Acon. nap. | gr. j |
| | Spongia usta | gr. j |
| | Hep. sulph. | gr. j |
| | Aquæ dest. | foz. viij |

M. S.—One teaspoonful every five or ten minutes, according to the severity of the case.

The sponge must be prepared as follows: Select a specimen that is not bleached, cut it into small pieces, and roast it until it can be readily reduced to powder, and you have all that you need in that direction. You may in some advanced cases require the following ointment:

| | | |
|---|---|---|
| ℞ | Ung. hyd. iod. rub . . . . . . . . . . | gr. v |
| | Vaseline . . . . . . . . . . . . . . . | oz. j |

But I seldom require it, as No. 1 formula is generally all that is necessary.

**Antifebrin—Some Useful Hints.**—Dr. Bryan writes to the Med. World: I wish to give your readers some hints as to the use of antifebrin which would have been invaluable to me a year ago.

1. *Don't give antifebrin and opiates at the same time or alternately* to the same patient. This will save you and your patients' friends a great deal of anxiety. If you must give opiates use some other febrifuge until you can drop the opiate.

2. Continue your antifebrin with quinine if you would avoid great depression.

3. Give antifebrin in the mucilage of elm bark to women and children.

4. If you have an intelligent nurse leave a thermometer and have the drug administered according to the requirements of the case; this is especially needful in typhoid fever.

5. Four grain doses repeated every hour till three doses are taken, the patient keeping the recumbent posture, will subdue nervous headache or sick headache.

6. Except in very high pyrexiæ the third or fourth hour interval will suffice and four grains is a sufficient dose.

7. In obstinate and high fever use three grains of antifebrin and six or eight grains of quinine together every hour till the temperature falls to 100° F.; this will hold the temperature down about eighteen hours.

8. Never use antifebrin and spts. eth. nit. dulc. in combination. This sounds dogmatic, but I do not wish to take the time or to infringe upon your space to give reasons.

---

**Summary of the General Treatment of Syphilis.**—1. Syphilis is a chronic disease, and requires a long continued course of treatment.

2. Mercurial treatment should be commenced as early as possible in cases with marked onset; in slighter forms somewhat later.

3. Mercurial treatment should be continued for two or three months. Then the iodide should be given for two months.—*Medical Digest.*

---

**Hot Water in Epistaxis.**—Epistaxis can be frequently arrested by immersion of the part in hot water; the following also can be used: ℞. Acid. tannic.; acid galic., aa, ʒ j. M. Sig. Insufflate in the nostrils. (SAJOUS.)

---

**Peach Leaf Poultice.**—Peach leaves pounded to a pulp and applied to bruise or wound from a rusty nail, or simple cut, it is stated will give immediate relief.

